# Case report: Eosinophilic pneumonia associated with vedolizumab therapy in a patient with ulcerative colitis

**DOI:** 10.3389/fmed.2022.942237

**Published:** 2022-08-05

**Authors:** Wanwan Zhu, Tianhao Zhao, Jun Wei, Damin Chai, Cancan Zhao, Yu Zhu, Min Deng

**Affiliations:** ^1^Department of Gastroenterology, The First Affiliated Hospital of Bengbu Medical College, Bengbu, China; ^2^Department of Pathology, The First Affiliated Hospital of Bengbu Medical College, Bengbu, China; ^3^Department of Radiology, The First Affiliated Hospital of Bengbu Medical College, Bengbu, China

**Keywords:** vedolizumab, eosinophilic pneumonia, adverse drug reactions, ulcerative colitis, case report

## Abstract

Extraintestinal manifestations are common in patients with inflammatory bowel disease, while respiratory involvement is less common. Vedolizumab is a new class of anti-integrin biological agents approved for treating inflammatory bowel disease. In this report, we present the case of a 38-year-old patient with ulcerative colitis for 7 years who developed cough, fever, and pulmonary infiltrates after taking vedolizumab. There was a spontaneous improvement in clinical symptoms and radiological abnormalities after discontinuing vedolizumab and introducing steroids. Despite the rarity of vedolizumab-induced eosinophilic pneumonia, the case reports indicate that patients with unexplained respiratory symptoms that are taking vedolizumab should be fully contemplated.

## Introduction

Ulcerative Colitis (UC) is a chronic disease affecting the colon and rectum and usually results in diarrhea with blood and mucus discharge; it may cause structural damage to the intestines and disability. Conventional therapies include aminosalicylates, corticosteroids, and immune modulators (1). In patients who fail conventional maintenance therapy, biologic treatments such as anti-tumor necrosis factor (anti-TNF), anti-integrin antibodies, a biologic agent against the p40 subunit of interleukin-12/23, Janus kinase (JAK) inhibitor, and a sphingosine-1-phosphate (S1P)–receptor modulator are usually recommended (2, 3). Vedolizumab (VDZ), a humanized monoclonal antibody α4β7 integrin receptor antagonist, is used for adults with moderately to severely active ulcerative colitis and Crohn’s disease who have failed at least one conventional therapy or intolerance to a TNF-a inhibitor therapy (4, 5), and its effectiveness and general safety have been established (6). Eosinophilic pneumonia (EP) is an inflammatory lung disease characterized by the infiltration of eosinophils into the alveolar region and interstitium of the lung, the gold standard for the diagnosis of it is lung biopsy or bronchial lavage fluid examination (7).

Here, we report a case of eosinophilic pneumonia in a patient with UC that coincided with the beginning of VDZ. Likewise, we review the literature on association between VDZ and eosinophilic pneumonia.

## Case presentation

Our patient was admitted to our hospital after experiencing a week of fever and cough on 8 September 2021. Seven years prior to admission, she was diagnosed with UC following recurrent abdominal pain, diarrhea, and mucopurulent hematochezia. Multiple conventional therapies had been used to treat the patient, including oral and enema mesalazine, oral corticosteroids, and intestinal flora adjustment. Despite these treatments, she still experienced frequent flare-ups. As a result of the failure of conventional therapies and the presence of ongoing active disease, it was decided to begin her on vedolizumab treatment (induction and maintenance) and to continue her mesalazine treatment. After evaluating the condition, she was initiated on VDZ and received three doses of 300 mg intravenous infusions on weeks 0, 2, and 6. A month after the third dose of VDZ therapy, she was admitted to the hospital with cough and fever. There was no significant medical history except for a “cesarean section” surgery 10 years ago. Besides, she was negative for allergic and respiratory diseases.

**FIGURE 1:**
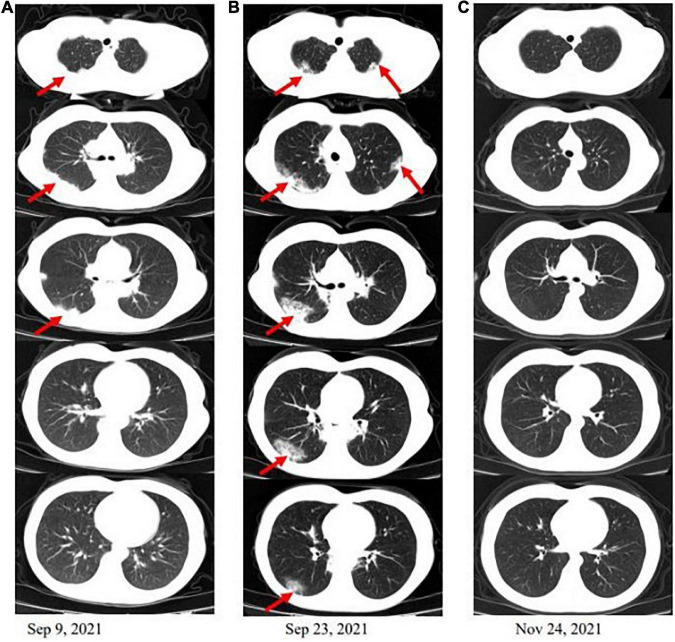
Computed tomography (CT) scan of the chest. **(A)** There are lesions of infiltrates under the pleura of the right lung when the patient is admitted. **(B)** After 2 weeks of antibiotic therapy, countercheck thoracic CT shows that pulmonary infiltrates are not significantly absorbed and appear to spread to the left lung and lower in the right. **(C)** The infiltrations under the pleura in both lungs were basically absorbed after a mouth of corticosteroid therapy.

On admission, she was normothermic (37°C) and had a blood pressure of 100/60 mmHg, a heart rate of 80 beats per minute, and a respiratory rate of 14 times per minute. The physical examination revealed moderate emaciation (BMI 17.6), crackles in the lower pulmonary fields could be heard, and old surgical scars on the abdomen could be seen. Other than that, the physical examination was unremarkable. The result of the laboratory work showed an increased eosinophil count of 1.4 × 10^9/L (normal 0.02–0.52 × 10^9/L), as well as small cell hypochromic anemia (Hb 85 g/L, HCT.306 L/L, and MCV 65 fL). Serum C-reactive protein level was 85.3 mg/L (normal range × 6 mg/L), and erythrocyte sedimentation rate was 46 mm/h (normal range × 20 mm/h). The fecal occult blood test showed 0-1 white blood cells per high-power field in occult blood. No Salmonella, Shigella, Campylobacter, or parasites were detected in stool samples. Respiratory and allergic diseases were not found. The bacterial, mycobacterial, and fungal cultures proved negative. Extensive microbiology assays (sputum culture, galactomannan and glucan testing, aspergillus and mycoplasma antibodies, hepatitis B virus, and *Treponema pallidum* antibodies) identified no infectious causes. The antinuclear antibody panel, ds-DNA antibodies, and immunoglobulin levels were not elevated to pathologic levels in the serologic examination. The tumor markers carcinoma embryonic antigen, alpha-fetoprotein, serum CA125, CA15-3, and CA19-9 were all within normal limits. Together, multiple microbiological cultures and tumor markers came up negative, effectively ruling out infection and neoplasm. The admission thoracic CT (Figure 1A) shows that there are lesions of infiltrates under the pleura of the right lung.

**FIGURE 2:**
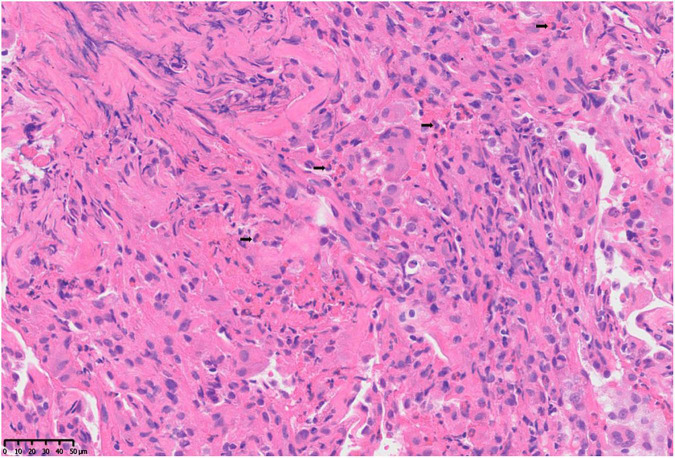
Pathological changes of lung tissue: microscopic finding of the right lower lung shows a heavy eosinophilic infiltration, > 20 eosinophils per high power field (H and E, ×400).

After 2 weeks of antibiotic therapy (levofloxacin 400 mg/day, cefotaxime 600 mg/day), the thoracic CT (Figure 1B) showed that the lesions of infiltrates were not significantly absorbed, which demonstrated that the symptoms of fever and cough may not be caused by infection. The retesting blood routine showed that eosinophil count continued to be increased at 4.02 × 10^9/L (normal 0.02–0.52 × 10^9/L) and was higher than that on admission. Then, she decided to go to a superior hospital for treatment. Endobronchial Ultrasound (EBUS) shows that exploration of the dorsal segment of the lower right lobe and low echo area, from which puncture it to get lung tissue. The pathological results showed that the interstitial fibrous tissue was hyperplasia, and there were many lymphocytes, plasma cells, and eosinophils infiltrated in the observation area (about 20 cells/HP in a dense area). No positive bacteria were detected by special staining (Figure 2).

**FIGURE 3:**
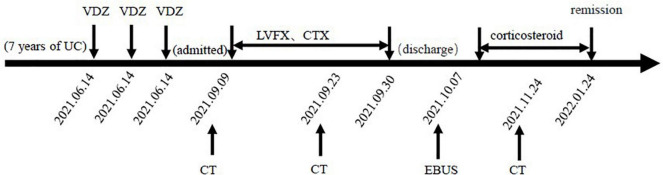
Changes in the condition after vedolizumab (VDZ) therapy. VDZ, vedolizumab; LVEF, levofloxacin; CTX, cefotaxime; CT, computed tomography; EBUS, endobronchial ultrasound.

Because of persistent high eosinophilic count and productive cough, which did not respond well to the antibiotic therapy, the patient was discontinued on VDZ, started on steroids, and showed significant improvement clinically. Three months later, the patient was asymptomatic, and the thoracic CT scan showed that the pulmonary infiltrations had resolved (Figure 1C). The patient has been discharged home and regularly followed up regularly in the gastroenterology and respiratory clinic. For changes in the condition after VDZ therapy, refer to Figure 3.

## Discussion

Inflammatory bowel disease (IBD) is often associated with symptoms outside the gastrointestinal tract but is less likely to involve the respiratory system. Extraintestinal complications associated with IBD can be divided into those induced by the disease and those induced by its treatment (8). Eosinophilic pneumonia (EP), whose symptoms include fever, cough, sputum production, and difficulty breathing, is a heterogeneous group of disorders characterized by pulmonary tissue and/or peripheral blood eosinophilia. Peripheral blood eosinophilia is usually the primary clue to the diagnosis of eosinophilic lung disease, BALF is an important means, and lung biopsy is the “gold standard” for diagnosis. Its etiology is unclear and may be related to parasites, fungal infections, drugs, and chemical toxicity exposure (9). A common cause of EP is drug toxicity, among the most common medications involved are antibiotics, non-steroidal anti-inflammatory drugs, and serotonin reuptake inhibitors. Usually, the problematic drug should be removed, and in some instances, systemic corticosteroids may be mandatory required.

The diagnosis of eosinophilic pneumonia in our case was based on persistent high eosinophilic levels and productive cough that were insufficiently responsive to antibiotic therapy, and the combination of imaging and therapeutic outcomes did not support the diagnosis of an infection. However, peripheral blood eosinophilia may be associated with UC in an active disease or use of mesalazine (10, 11). After our investigation, the patient’s previous eosinophils were normal, both during the active and plateau phases of UC; there was no such pulmonary manifestation before using VDZ, and the possibility of considering the UC disease itself with pulmonary abnormalities was small. Besides, because our patient had been taking mesalazine during the course of the disease l and periods of remission, EP caused by mesalazine was also excluded. Drug-associated eosinophilic pneumonia is considered in combination with the patient’s medication history and time of symptom onset. The score on the Adverse Drug Reaction Probability Scale was 4, signifying a possible adverse drug reaction (12).

In addition to our case, we only identified two cases of eosinophilic complications associated with VDZ treatment on the databases of Medline. One is the case of a 22-year-old female with UC who developed eosinophilic bronchial asthma a month after using VDZ; her pulmonary symptoms improved, and peripheral eosinophilia showed partial improvement a year after discontinuing the drug (13). The other is the case of a 49-year-old smoking man with a history of pruritus nodosa who developed chronic EP after VDZ treatment of UC. Pneumonia symptoms did not respond to corticosteroids, and the patient was treated with mepolizumab; the symptoms improved within 1 week. Pulmonary opacities improved in 6 months of therapy (14). In all 3 cases, BAL or EOS or surgical biopsy had evidence of pulmonary eosinophilia or eosinophilic bronchial asthma (Table 1).

**TABLE 1:** Characteristics of patients with eosinophilic pneumonia associated with vedolizumab (VDZ).

Source	Age/sex	Smoker	underlying disease	Symptoms	Duration of VDZ	EOS	BAL	Biopsy	VDZ discontinuation	Steroid treatment	Outcome
Current case report (2022)	38/F	No	UC	Cough, fever	6 weeks	Yes	–	Heavy eosinophilic infiltration	Yes	Yes	Improvement
Dulaney et al. (13)	22/F	No	UC	Dyspnea	4 weeks	Yes	–	–	Yes	Yes	Improvement
Lawrence and Klings (14)	49/M	Yes	UC	Cough, dyspnea	–	No	Negative	Consistent with CEP	No	Yes	Improvement after adding mepolizumab

EOS, eosinophilia; BAL, bronchoalveolar lavage; UC, ulcerative colitis; –, no data available or examination was not performed.

The mechanism of VDZ-associated EP may include obstruction of VDZ-associated cells in the gastrointestinal tract, allowing for immune effector cells to spread to external intestinal sites (15). As an “intestine-specific” biologic for IBD therapy. Vedolizumab interferes the adhesion of the α4β7 integrin with its ligand, mucosal addressin cell adhesion molecule 1 (MAdCAM-1). Cells expressing MAdCAM-1, which is mainly expressed in intestinal tissues, are preferentially localized in intestinal tissues (16). However, α4β7-expressing cells circulate extensively in our body, and VDZ-related extraintestinal manifestations occur occasionally (17, 18). Other possibilities include expulsion of inflammatory cells from the inflamed intestine after VDZ treatment and upregulation of alternative integrins such as α4β1, leading to changes in the pattern of cell localization to the intestines and external intestinal sites. Lissner et al. analyzed integrin expression in whole blood samples from patients and showed neutralization of α4β7 on naive and memory T lymphocytes in anticipation of VDZ treatment. Interestingly, they observed simultaneous increases in other lung integrins (β1 integrin) leading to behavioral changes in leukocyte migration to organs outside the gut (19). Alternatively, VDZ-induced EP may be a non-IgE-mediated hypersensitivity reaction (20), or may reflect drug-related induction of eosinophils through mechanisms that are poorly understood or a heterogeneous response to drugs (21).

## Conclusion

In brief, we report a case of eosinophil pneumonia and review two other cases of pulmonary hypereosinophilia in patients treated with VDZ. In all the patients, the pulmonary symptoms partially improved and subsided by VDZ withdrawal or initiation of corticosteroids, which suggests that there might exist a potential association between the use of VDZ and pulmonary disease. It may support the emerging hypothesis that gut selective biologics can identify upregulation of certain pro-inflammatory factors, ultimately leading to development of lung disease. Future research can be in clinical, epidemiological and examine the potential link between the two mechanisms level, and may further understand resistance integrin parenteral disease induced by eosinophils after treatment, the relevant experts to pharmacology study the mechanism of action of anti-integrin biological preparation provides more thinking. Physicians must maintain a high degree of suspicion for the development of pulmonary disease in the context of IBD so that appropriate treatment can be administered early to avoid complications.

## Data availability statement

The original contributions presented in the study are included in the article/supplementary material, further inquiries can be directed to the corresponding author/s.

## Ethics statement

Written informed consent was obtained from the individual(s) for the publication of any potentially identifiable images or data included in this article.

## Author contributions

MD, JW, and YZ were involved in the patient’s clinical treatment. CZ and DC contributed to the diagnosis of the study. DC analyzed the pathological images. CZ analyzed the computed tomography images. WZ integrated all information and wrote the manuscript. TZ and WZ participated in the follow-up process. MD provided critical guidance and revisions for WZ throughout the writing process. All authors contributed to the article and approved the submitted version.

## References

[1] Ungaro R Mehandru S Allen PB Peyrin-Biroulet L Colombel JF. Ulcerative colitis. *Lancet.* (2017) 389:1756–70. 10.1016/S0140-6736(16)32126-2 27914657 PMC6487890

[2] Yamamoto-Furusho JK Parra-Holguín NN. Emerging therapeutic options in inflammatory bowel disease. *World J Gastroenterol.* (2021) 27:8242–61. 10.3748/wjg.v27.i48.8242 35068868 PMC8717021

[3] Baumgart DC Le Berre C. Newer biologic and small-molecule therapies for inflammatory bowel disease. *N Engl J Med.* (2021) 385:1302–15. 10.1056/NEJMra1907607 34587387

[4] Feagan BG Rutgeerts P Sands BE Hanauer S Colombel JF Sandborn WJ Vedolizumab as induction and maintenance therapy for ulcerative colitis. *N Engl J Med.* (2013) 369:699–710. 10.1056/NEJMoa1215734 23964932

[5] Sandborn WJ Feagan BG Rutgeerts P Hanauer S Colombel JF Sands BE Vedolizumab as induction and maintenance therapy for Crohn’s disease. *N Engl J Med.* (2013) 369:711–21. 10.1056/NEJMoa1215739 23964933

[6] Sandborn WJ Baert F Danese S Krznarić Ž Kobayashi T Yao X Efficacy and safety of vedolizumab subcutaneous formulation in a randomized trial of patients with ulcerative colitis. *Gastroenterology.* (2020) 158:562-572.e12. 10.1053/j.gastro.2019.08.027 31470005

[7] Cottin V. Eosinophilic lung diseases. *Clin Chest Med*. (2016) 37:535–56. 10.1016/j.ccm.2016.04.015 27514599

[8] Ott C Schölmerich J. Extraintestinal manifestations and complications in IBD. *Nat Rev Gastroenterol Hepatol*. (2013) 10:585–95. 10.1038/nrgastro.2013.117 23835489

[9] Allen J Wert M. Eosinophilic pneumonias. *J Allergy Clin Immunol Pract*. (2018) 6:1455–61. 10.1016/j.jaip.2018.03.011 29735405

[10] Franco AI Escobar L García XA Van Domselaar M Achecar LM Luján DR Mesalazine-induced eosinophilic pneumonia in a patient with ulcerative colitis disease: A case report and literature review. *Int J Colorectal Dis.* (2016) 31:927–9. 10.1007/s00384-015-2318-3 26189026

[11] Click B Anderson AM Koutroubakis IE Rivers CR Babichenko D Machicado JD Peripheral eosinophilia in patients with inflammatory bowel disease defines an aggressive disease phenotype. *Am J Gastroenterol.* (2017) 112:1849–58. 10.1038/ajg.2017.402 29112200

[12] Naranjo CA Busto U Sellers EM Sandor P I Roberts EA Janecek E A method for estimating the probability of adverse drug reactions. *Clin Pharmacol Ther.* (1981) 30:239–45. 10.1038/clpt.1981.154 7249508

[13] Dulaney D Dave P Walsh S Mehandru S Colombel JF Agrawal M. Noninfectious pulmonary complications associated with anti-integrin therapy: A case report and systematic review of the literature. *Inflamm Bowel Dis.* (2022) 28:479–83. 10.1093/ibd/izab212 34427639 PMC9122753

[14] Lawrence R Klings ES. Management of chronic eosinophilic pneumonia with mepolizumab. *Am J Respirat Crit Care Med.* (2019) 199:A1522. 10.1164/ajrccm-conference.2019.199.1_MeetingAbstracts.A1522

[15] Abu Shtaya A Cohen S Kogan Y Shteinberg M Sagool O. Crohn’s disease with atypical extra-intestinal manifestations developing under treatment with vedolizumab. *Eur J Case Rep Internal Med.* (2021) 8:002265. 10.12890/2021_002265 33768073 PMC7977061

[16] Wyant T Fedyk E Abhyankar B. An overview of the mechanism of action of the monoclonal antibody vedolizumab. *J Crohns Colitis.* (2016) 10:1437–44. 10.1093/ecco-jcc/jjw092 27252400

[17] Pugliese D Privitera G Schepis T Larosa L Onali S Scaldaferri F Drug-related pneumonitis in patients receiving vedolizumab therapy for inflammatory bowel disease. *Clin Gastroenterol Hepatol.* (2021) 20:e1483–7. 10.1016/j.cgh.2021.08.041 34478878

[18] Diaz LI Keihanian T Schwartz I Bin Kim S Calmet F Alejandra Quintero M Vedolizumab-induced de novo extraintestinal manifestations. *Gastroenterol Hepatol (N Y).* (2020) 16:75–81. 34035705 PMC8132677

[19] Lissner D Glauben R Allers K Sonnenberg E Loddenkemper C Schneider T Pulmonary manifestation of Crohn’s disease developed under treatment with vedolizumab. *Am J Gastroenterol.* (2018) 113:146–8. 10.1038/ajg.2017.395 29311733

[20] Joshi SR Khan DA. Non-IgE-mediated drug hypersensitivity reactions. *Curr Allergy Asthma Rep.* (2021) 21:41. 10.1007/s11882-021-01018-7 34463914

[21] Roden AC Camus P. Iatrogenic pulmonary lesions. *Semin Diagnost Pathol.* (2018) 35:260–71. 10.1053/j.semdp.2018.03.002 29631763

